# Long-Distance Axial Trapping with Focused Annular Laser Beams

**DOI:** 10.1371/journal.pone.0057984

**Published:** 2013-03-07

**Authors:** Ming Lei, Ze Li, Shaohui Yan, Baoli Yao, Dan Dan, Yujiao Qi, Jia Qian, Yanlong Yang, Peng Gao, Tong Ye

**Affiliations:** State Key Laboratory of Transient Optics and Photonics, Xi'an Institute of Optics and Precision Mechanics, Chinese Academy of Sciences, Xi'an, China; University of Zurich, Switzerland

## Abstract

Focusing an annular laser beam can improve the axial trapping efficiency due to the reduction of the scattering force, which enables the use of a lower numerical aperture (NA) objective lens with a long working distance to trap particles in deeper aqueous medium. In this paper, we present an axicon-to-axicon scheme for producing parallel annular beams with the advantages of higher efficiency compared with the obstructed beam approach. The validity of the scheme is verified by the observation of a stable trapping of silica microspheres with relatively low NA microscope objective lenses (NA = 0.6 and 0.45), and the axial trapping depth of 5 mm is demonstrated in experiment.

## Introduction

Since its first demonstration in 1986 by Ashkin *et al.* [1], optical tweezers has been serving as a powerful tool for microscopic trapping and manipulating, providing a stimulus to many research fields, such as in physics [2], biology [3] and colloid [4].

In an optical tweezers, stable trapping requires that the gradient force overcomes the scattering force, the former of which depends on the gradient of the intensity of the focused fields and the latter increases with increasing energy flow. Generally, stably axial trapping is more difficult than lateral trapping because of the intensively axial scattering force exerted by the axial energy flow in the focal region. As a result, the reduction of the axial scattering force is a key factor in stably axial trapping. Different approaches to improving axial trapping efficiency have been demonstrated to date. For larger particles, the use of higher-order Laguerre-Gaussian (LG) beam modes can improve axial trapping efficiency [5], [6]. But it should be noted that, although the LG beam is a hollow beam in intensity shape, it has a spiral phase distribution, which makes it have very different focusing property rather than other hollow beams with homogeneous phase distribution. Raktim Dasgupta et al. [7] utilized LG_01_ mode to trap silica microspheres at 200 µm axial distance. Rodrigo et al. [8], [9] demonstrated 3D trapping with long working distance via counter propagating light fields. Recently, radially polarized beam is shown to produce a vanishing axial Poynting vector component on the optical axis in the focal region, leading to a higher axial trapping efficiency in comparison with the linearly polarized beam or circularly polarized beam [10]–[14]. Double-ring radially polarized beams can further make the enhancement of the axial trapping efficiency [15]. Bowman [16] and Thalhammer et al. [17]. demonstrated a “macro-tweezers” approach, and the optical mirror trap was created after reflection of two holographically shaped collinear beams on a mirror. Although all these approaches are promising, their implementation demand some special optical elements such as spiral phase plate or spatially varying retarders, which limits their applicability. Ashkin [18] predicated that the use of an obstructed beam could increase the axial trapping efficiency of a dielectric particle since the annular intensity distribution enhanced the contribution of rays with a large angle of convergence, that would decrease the axial scattering force. Gu and Morrish [19] proved that Mie metallic particles were axially trapped with a centrally obstructed Gaussian (TEM_00_-mode) beam focused by a high NA objective lens. Commonly, an opaque disk is used in the obstructed beam approach. This allows only those rays converging at large angles to be focused. The maximal axial trapping efficiency increases with the size of the center obstruction, but most of the incident light will be lost in this geometry. In addition, most of the axial trapping techniques developed so far utilize objective lenses with high numerical aperture (NA>1), which enable higher spatial resolution and axial trapping efficiency. However, high NA objective lenses are generally designed with a very short working distance (typically less than 0.2 mm), that means in many cases that the available space is too small to move the sample axially. High NA objectives also suffer from spherical aberrations when used for imaging in aqueous solutions. The spherical aberration introduced by the refractive difference between glass and water will produce a degradation of the imaging performance and inevitably limit the trapping depth in the aqueous medium [20], [21].

In this paper, we investigate theoretically the optical trapping efficiency on dielectric particles and make comparison of the axial and lateral trapping efficiencies for different widths of the annular beams by using vectorial diffraction theory. Long working distance objectives from Nikon Inc. and Edmund Optics Inc. (working distance are 8 mm and 13 mm, respectively) are used to focus the parallel annular laser beam to near diffraction-limited focal spot and realize the three-dimensional optical trapping. The parallel annular beam is generated by a telescopic pair of axicon lenses with transmittance of nearly 100%. The axial trapping depth of 5 mm is realized in the experiment. Theoretical calculations and experimental results are in good agreement.

## Theoretical Calculation

In this section, we present the numerical results to show how an annular beam improve the axial trapping efficiency. The optical trapping force ***F*** on the particle can be calculated by vectorial diffraction theory based on the electromagnetic scattering theory [11], [22]. Instead of using the force ***F***, we generally use a dimensionless quantity, trapping efficiency ***Q,*** to characterize how effective the optical trapping is. The trapping efficiency ***Q*** is defined, in component form, as
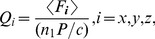
(1)where *P* is the power of the incident laser beam, *c* is the speed of light in vacuum, *n*_1_ is the refractive index of surrounding medium, and <*F_i_*> is the time-averaged trapping force. Throughout the paper, the wavelength is set to be λ = 491 nm, the refractive index of the particle is *n*_2_ = 1.5 and that of the surrounding medium is *n*_1_ = 1.33, the radius of the spherical particle is *a* = 2 µm. For the convenience of discussion, a parameter *d*, which can be regarded as the normalized width of the annular beams is introduced and defined as *d* = (*r*_2_−*r*_1_)/*r*_2_, where *r*_1_ and *r*_2_ are the inner radius and outer radius of the annular beam, respectively. It can be seen that with the increasing of the value of *d*, the width of the annulus increases accordingly.

**Figure 1:**
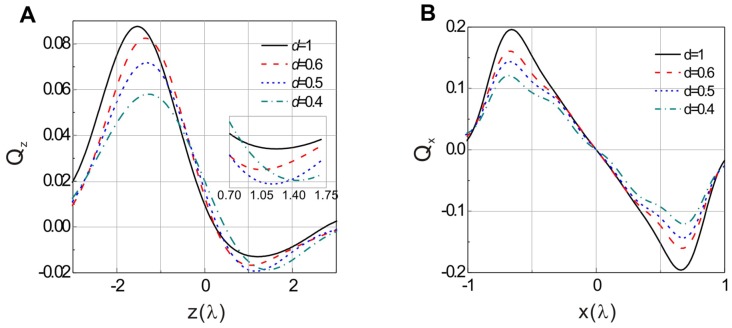
Calculated trapping efficiencies of a spherical particle by annular beams with different widths. **A.** Axial trapping efficiency, **B.** lateral trapping efficiency. The trapping wavelength is 491 nm, the sphere radius is 2 µm, and the numerical aperture of the objective is 0.6.

The axial trapping efficiency *Q*_z_ is calculated based on the vectorial diffraction theory [23], [24]. Figure 1 (a) shows the calculated curves of the axial trapping efficiencies of the annular beams with different *d* focused by a NA = 0.6 objective. For small values of *d*, the maximal backward trapping efficiency *Q*_max_ increases with increasing of *d*. This corresponds to the cases of *d* = 0.4 and 0.5. With further increasing of *d*, *Q*_max_, however, decreases, as can be seen for *d* = 0.6 and 1.0 curves. This implies that there is a critical value of *d*, say *d*_0_, for which *Q*_0max_ assumes its maximum. The occurrence of *d*_0_ is a result of the competition between the scattering force and the gradient force in the focal region. A annular with small values of *d* can offer a higher portion of rays with large converging angle of focusing, leading to a reduction of the scattering force. However, a smaller value of *d* also results in the extent of the axial focal depth, which will reduce the axial gradient force. Therefore, it is important to find an optimal value of *d* in practice. From Figure 1(a) we can see that the maximal backward axial trapping efficiency at the optimal annulus (*d* = 0.5) has an improvement up to 49.2% in comparison with the TEM_00_ beam (*d* = 1).

Figure 1(b) gives the lateral trapping efficiency for different values of *d*. All the while, the maximal lateral trapping efficiency falls with decreasing of the values of *d*. This is because that the gradient force is dominant due to the multiple refraction on the surface of the trapped dielectric particle [25], i.e., there will be much loss of lateral trapping force when *d* is smaller. However, the reduction of lateral trapping efficiency has little influence on stably lateral trapping in practice.

## Methods

**Figure 2:**
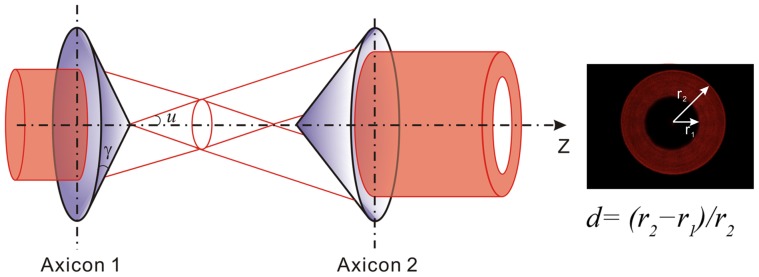
Axicon-pair for generation of parallel annular beam.

Axicon (conical lens) is a well known wavefront division optical element to transform a Gaussian beam into a Bessel beam, and has already found applications in optical trapping [26] and multifunctional darkfield microscopy [27]. Shao et al. [28] utilized a 3-axicon approach to generate a three-dimensional ring-shaped trap. We adopt here a telescopic pair of axicons to transform a Gaussian beam into a parallel annular beam. As shown in Figure 2, a collimated Gaussian beam incident on the base of the first axicon is deviated cylindrically toward the optical axis due to refraction. All deviated rays independently propagate along different directions and form a hollow cone beam, and then incident on the conical surface of the second axicon with a ring-shape of intensity distribution. Two axicons with the same open angle are arranged tip to tip to ensure the output beam behind the second axicon parallel to the optical axis. Adjusting the relative distance between the two axicons corresponds to changing the diameter of the output parallel annular beam [29]. By adjusting the diameter of the output parallel annular beam to overfill the back aperture of the objective len, a near diffraction-limited focus can be obtained. Considering the light transmittance, the open angle *γ* of the axicon is usually designed very small (less than 10 degree), giving a good approximate calculation of the divergence angle *u*≈(*n*−1)*γ*, where *n* is the refractive index of the axicon. We used two axicons from Thorlabs Inc. with the open angle *γ* = 5° and the refractive index *n* = 1.5. One of the advantages of using the axicon-pair geometry is the very high transmittance, which can be nearly 100% in theory. Considering the transmittance of the antireflection coating of the two axicons, the total conversion efficiency can still reach to 90% in practice.

**Figure 3:**
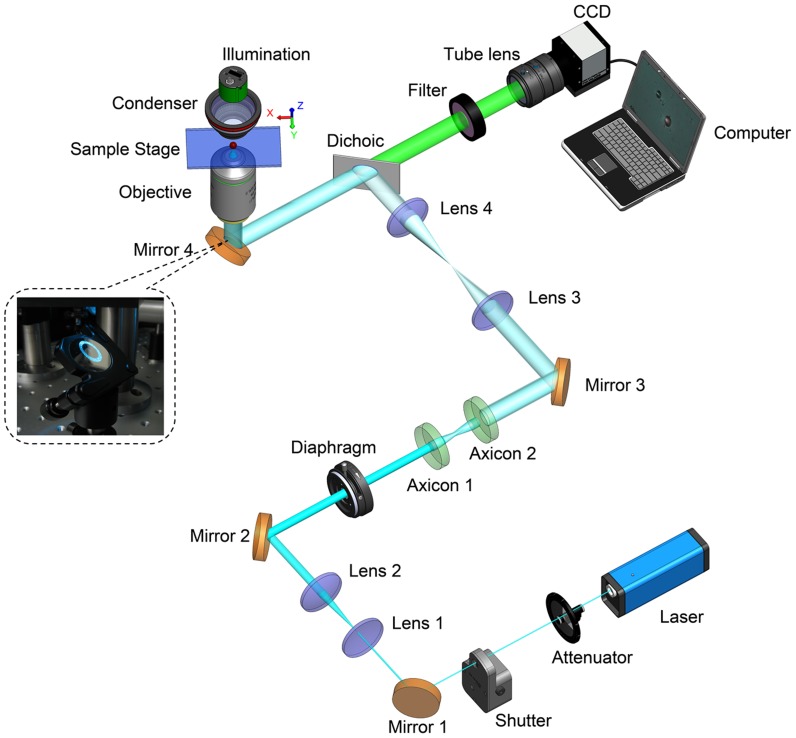
Experimental layout of the trapping system. The inset gives the annular beam shape captured on Mirror 4.

Figure 3 shows the experimental layout of our trapping system. A diode-pumped solid-state laser (Calypso 491, Cobolt AB Inc., Sweden) working at wavelength of 491 nm is expanded by a telescope formed by Lens 1 and Lens 2. After beam expanding, the collimated beam passes through the telescopic pair of axicon to be transformed into a parallel annular beam. The parallel annular beam then passes through another telescope comprised of Lens 3 and Lens 4, and then is reflected by a dichroic mirror (short-pass 475 nm) into the back aperture of the long working distance 20X objective (EO M Plan HR, NA0.6, 13 mm working distance, Edmund Optics Inc., USA) or a 20X objective (Plan Fluor, NA0.45, 8 mm working distance, Nikon Inc., Japan). Sliding the Lens 4 will slightly change the divergence of the annular shaped beam and ensure that it can be focused on the sample exactly. The sample is mounted on a motorized XYZ stage (MP-285, Sutter Instrument Inc., Canada) that can be driven either manually or by a programmable software interface. A USB CCD camera (DMK 41BU02, 1280×960 pixels, 4.65 µm×4.65 µm pixel size, The Imaging Source Europe GmbH, Germany) is employed to record the trapping process, with an lens (50 mm, F/1.8, Nikon Inc, Japan) serving as a tube lens. The CCD camera allows imaging speed as high as 30 fps at a resolution of 1280×960 pixels. A long-pass filter (long-pass 505,) is inserted in front of the CCD camera to block the trapping laser beam. The maximal laser power delivered on the sample is about 10 mW. Silica microspheres (4 µm in diameter, Polysciences Inc., USA) immersed in water in a sample chamber are used as test samples.

## Results and Discussion

**Figure 4:**
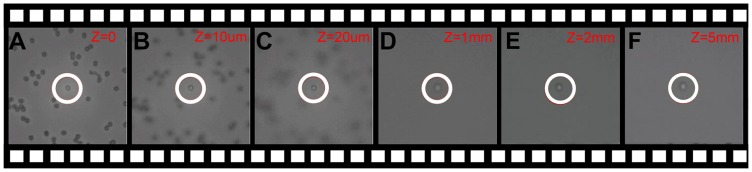
Trapping and moving a silica microsphere in 5 mm depth axially with the focused annular beam. **A–F** show the trapped microsphere at different axial planes. Using a long working distance microscope objective (20X/NA0.6) (Video S1).

The axial trapping force *F_z_* on a trapped silica microsphere suspended in water was measured by observing the maximal translation speed of the motorized stage at which the particle fell out of the trap. The force is calculated by the Stokes law *F* = 6*πηaν*, where *a* is the radius of a trapped particle, *ν* is the maximal translation speed, and *η*is the viscosity of the surrounding medium. The maximal axial trapping efficiency *Q_z,max_* is then calculated by Eq.(1). In the trapping experiment, the upward motion of the motorized stage is chosen, because the trap is weakest in that direction in the inverted microscope due to the opposing action between the scattering force and the gradient force. The experiment is repeated several times to reduce the error. The measured maximal axial trapping efficiency is 0.0104. Figure 4 (Video S1) demonstrates actually an axial movement of a trapped silica bead in 5 mm depth with a 20X/NA0.6 objective. The axial movement of the particle is controlled by a motorized stage with resolution of 200 nm, and the moving speed and position are controllable with the software. Figure 4A–F show the trapped bead at different axial planes. According to the working distance of the microscope objective, the maximal axial trapping depth can theoretically reach up to 13 mm.

**Figure 5:**
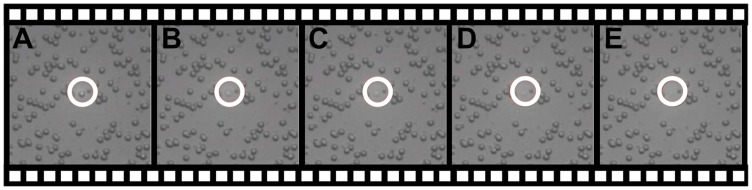
Pushing a silica microsphere out of the focus with the focused TEM_00_ beam. Using a long working distance microscope objective (20X/NA0.6). (Video S2).

As expected, the trapping force caused by the TEM_00_ Gaussian beam focused by the same objective failed to trap the same particle, and always pushed the particle out of the focus. This process is demonstrated in Figure 5 (Video S2).

**Figure 6:**
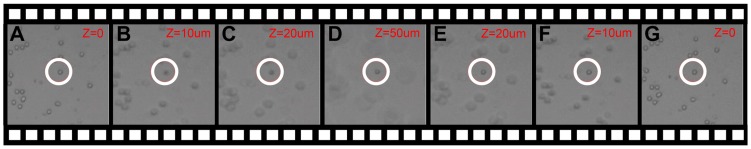
Trapping and moving a silica microsphere axially by an objective lens with much lower NA(0.45). **A–D** moving upward, **E–G** moving backward. (Video S3).

We also demonstrate that it is even possible to trap the particles by an objective with much lower NA(0.45), as shown in Figure 6 (Video S3).

**Figure 7:**
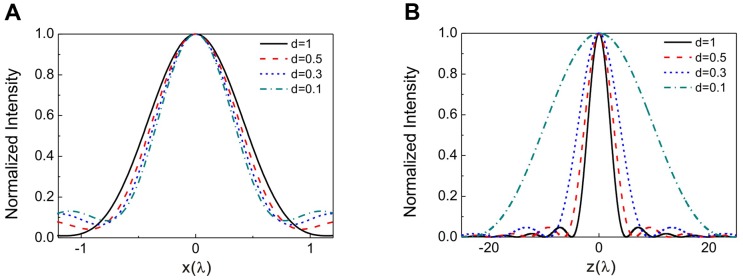
Simulated intensity distributions of the focused annular beam with different widths. **A.** Lateral intensity distribution, **B.** axial intensity distribution. The numerical aperture of the objective is 0.6.

Gu et al. [19] proposed a method for three-dimensional optical trapping of metallic Mie particles using an obstructed laser beam based on the geometrical optics model. The maximal axial trapping efficiency increases with increasing of the size of the center obstruction in their calculation. However, the geometrical optics model ignores the light intensity distribution in the focal region, and can only be employed as a good approximation to compute the force on particles which are much larger than the wavelength of the incident light. We tried decreasing the width of the annular beam to observe the impact on the axial trapping efficiency, but contrary to the calculation based on geometrical optics model, the axial trapping efficiency will decrease as the annular width keeps decreasing after the optimal value *d = 0.5*. We think this is because that the width of the annular beam has an impact on the intensity distribution near the focal region. Figure 7 gives the simulated lateral and axial intensity distributions for the annular beam with different widths focused by a NA = 0.6 objective. It can be seen that the lateral intensity distribution keeps almost unaltered with different widths. While for axial direction, the thin annular light field will lead to the focus to be stretched a lot along the axial direction, thereby reducing the axial trapping efficiency. This phenomenon was also mentioned by Kitamura et al. [30] The theoretical calculation of optical trapping force in our simulation is based on the vectorial diffraction theory, which provides a more rigorous solution of the scattering field and is not limited by the size and shape of particles in contrast with the geometrical optics model.

Trapping stiffness is a common parameter to quantify the stability of an optical tweezers system. It can be measured and calculated by using the probability distribution method. Generally, there are two main routes to measure the motion of trapped particles, i.e., quadrant photodiode (QPD) [31] and video-based particle tracking [32]. Due to the fluctuation of trapped particles is normally in the order of nanometers, such measurement typically needs oil-immersion objective with high magnification. In our presented system, a 20X objective is used to trap 4 µm-diameter particles. The resolution of our imaging system is 230 nm/pixel approximately, which is barely enough for the position measurement algorithm. From our trapping videos, we just estimate that the lateral stiffness of our tweezers system is about 3×10^−6^ N/m @ 20 mW. For integrity, we also applied a trapping force calibration technique as described above. The measured maximal axial trapping efficiency is consistent with the theoretical calculation.

Optical trapping of using large NA objectives has some limitations such as extremely short working distance, narrow field of view, and tight focusing with high power density, which might cause heating and optical damage to the biological specimen. In some applications, a large field of view and long working distance are highly desirable. Long working distance objectives are particularly useful for industrial inspection such as wafer probing and flaw detection. The utilization of such kind of objective in optical trapping may open the possibility of exploring large volume with optical tweezers. Due to the long distance imaging capability, the axicon-pair-based optical tweezers could also be a useful tool in various biological researches, for example, examining specimens in vitro through thick glass walls, where the objective lens must be protected against environmental hazards such as heat, vapors, and volatile chemicals by a thick coverslip.

## Conclusion

We have proposed a long distance axial trapping approach with focused annular laser beam based on a telescopic pair of axicon. The optical trapping efficiencies on dielectric particles for different widths of the annular beams have been calculated by using vectorial diffraction theory. By trapping silica microspheres suspended in water, we have demonstrated that the system has the advantages of higher efficiency and longer trapping range over the conventional axial trapping geometry.

## Supporting Information

Video S1 **Trapping and moving a silica microsphere in 5 mm depth axially with the focused annular beam.**
(MOV)

Video S2 **Pushing a silica microsphere out of the focus with the focused TEM_00_ beam.**
(MOV)

Video S3 **Trapping and moving a silica microsphere axially by an NA 0.45 objective lens.**
(MOV)
